# Therapeutic Approaches for Patients with Cystic Fibrosis Not Eligible for Current CFTR Modulators

**DOI:** 10.3390/cells10102793

**Published:** 2021-10-19

**Authors:** Isabelle Fajac, Isabelle Sermet

**Affiliations:** 1 AP-HP. Centre—Université de Paris, Hôpital Cochin, Centre de Référence Maladie Rare-Mucoviscidose, 75014 Paris, France; 2 Faculté de Médecine, Université de Paris, 75006 Paris, France; isabelle.sermet@aphp.fr; 3 INSERM U 1151, Institut Necker Enfants Malades, 75015 Paris, France; 4 AP-HP. Centre—Université de Paris, Hôpital Necker Enfants Malades, Centre de Référence Maladie Rare-Mucoviscidose, 75015 Paris, France

**Keywords:** cystic fibrosis, CFTR modulators, readthrough agents, RNA therapy, gene therapy, gene editing, cell-based therapy

## Abstract

Cystic fibrosis is a severe autosomal recessive disorder caused by mutations in the cystic fibrosis transmembrane conductance regulator (*CFTR*) gene encoding the CFTR protein, a chloride channel expressed in many epithelial cells. New drugs called CFTR modulators aim at restoring the CFTR protein function, and they will benefit many patients with cystic fibrosis in the near future. However, some patients bear rare mutations that are not yet eligible for CFTR modulators, although they might be amenable to these new disease-modifying drugs. Moreover, more than 10% of *CFTR* mutations do not produce any CFTR protein for CFTR modulators to act upon. The purpose of this review is to provide an overview of different approaches pursued to treat patients bearing mutations ineligible for CFTR modulators. One approach is to broaden the numbers of mutations eligible for CFTR modulators. This requires developing strategies to evaluate drugs in populations bearing very rare genotypes. Other approaches aiming at correcting the *CFTR* defect develop new mutation-specific or mutation-agnostic therapies for mutations that do not produce a CFTR protein: readthrough agents for nonsense mutations, nucleic acid-based therapies, RNA- or DNA-based, and cell-based therapies. Most of these approaches are in pre-clinical development or, for some of them, early clinical phases. Many hurdles and challenges will have to be solved before they can be safely translated to patients.

## 1. Introduction

Cystic fibrosis is the most common autosomal recessive disorder in the Caucasian population [1]. It affects approximately 90,000 individuals worldwide, and it is caused by mutations in the cystic fibrosis transmembrane conductance regulator (*CFTR*) gene [1]. The *CFTR* gene encodes the CFTR protein, which is mainly a chloride channel expressed in epithelial cells. Cystic fibrosis is a multi-system disease affecting organs and tissues wherein CFTR is expressed. The most common clinical features associate exocrine pancreatic insufficiency and bronchiectasis with chronic airway infection leading to respiratory failure and premature death. Cystic fibrosis treatment has long been based on symptom-based therapies focusing on compensating exocrine pancreatic insufficiency with pancreatic enzymes and on slowing lung disease progression with airway clearance techniques and antibiotic therapy [2]. Since the cloning of the *CFTR* gene in 1989 and the subsequent growing knowledge of the CFTR protein’s maturation, structure and function, the development of drugs correcting the basic defect in cystic fibrosis has been a major goal. Because the primary cause of morbidity and mortality in cystic fibrosis is due to progressive lung destruction, most new treatment approaches have targeted the airway epithelium and the lung disease. However, with local lung administration, other organs affected by the disease remain untreated. New drugs called CFTR modulators aim at restoring CFTR protein function. They have been developed in the last decade and they will benefit around 80% of patients with cystic fibrosis in Europe [3,4]. Once CFTR modulators can be given early in life, the even partial restoration of CFTR function will have a major impact on cystic fibrosis disease features and course throughout life. In Europe, around 20% of patients with cystic fibrosis are not currently eligible for CFTR modulators. Among these patients, some bear rare mutations that might be amenable to CFTR modulators and new ways of evaluating drugs in very scarce populations are needed. CFTR modulators require a CFTR protein to act upon, and among the 2000 *CFTR* mutations that have been identified, more than 10% do not produce any CFTR protein (cystic fibrosis mutation database http://www.genet.sickkids.on.ca/ accessed on 15 September 2021). These are all the mutations that include nonsense *CFTR* mutations (also called stop or premature termination codon (PTC) mutations), frame-shift mutations, large deletions and insertions and splice-site mutations causing frame-shifts, which often introduce a PTC. This review summarizes these different approaches targeting the CFTR gene, ARN or protein undertaken to treat patients bearing mutations that are not eligible for CFTR modulators. 

## 2. Broadening the Numbers of Mutations Eligible for CFTR Modulators

CFTR modulators were evaluated in clinical trials enrolling patients with cystic fibrosis bearing well-described and quite common mutations. Some rare mutations leading to a non-truncated CFTR protein might be amenable to CFTR modulators. However, these are less common mutations, and it is difficult to conduct clinical trials in very limited populations. Many preclinical models are being developed and assessed for their reliability and validity to predict individual outcomes from current and emerging CFTR modulators [5]. These are heterologous cell lines or patient-derived materials, such as nasal cell cultures or organoids, of which intestinal organoids are so far the most studied. In 2017, the Food and Drug Administration expanded the label of the first licensed CFTR modulator, ivacaftor, to include additional rare mutations [6]. This was based on in vitro assay data demonstrating increased chloride ion transport across cells in response to ivacaftor. This was groundbreaking from a regulatory perspective as no clinical data were required to expand the label. This approach was renewed for subsequent CFTR modulators (see Vertex press release of Dec 20, 2020), but it was not adopted by the European Medicines Agency. New ways of evaluating drugs in very scarce population need to be found and agreed on with regulatory bodies. This is the ultimate goal of the European initiative called HIT-CF (https://www.hitcf.org/ accessed on 15 September 2021): individual responses to drug candidates are evaluated on intestinal organoids grown from patients with cystic fibrosis carrying rare mutations. These individual responses will allow researchers to select and invite patients to participate in a clinical trial to study the efficacy and safety of the tested drug candidates. A high correlation between the in vitro effect of CFTR modulators on intestinal organoids and clinical responses has been shown, and this supported off-label treatment in some patients [7]. Primary nasal cells, which recapitulate the respiratory epithelium, are also used to select responsive variants. Whether they may be used to select responders in patients with the same genotype is currently being investigated [8].

## 3. Readthrough Agents for Nonsense Mutations

Some mutations called nonsense mutations convert a codon originally coding for an amino acid to one of the three termination codons (UAA, UAG or UGA), resulting in a PTC in the protein-coding sequence [9]. This PTC induces a premature termination of translation and produces truncated nonfunctional proteins that are readily degraded [10]. Moreover, transcript levels are decreased because of nonsense-mediated RNA decay (NMD), a surveillance mechanism that detects and degrades PTC containing transcripts, thus preventing the synthesis of truncated proteins [11]. Nonsense mutations account for around 10% of all cystic fibrosis mutations (cystic fibrosis mutation database http://www.genet.sickkids.on.ca/ accessed on 15 September 2021) and usually result in severe cystic fibrosis disease [12]. 

Various preclinical studies provide evidence that the translational readthrough of PTCs can be promoted pharmacologically by decreasing the fidelity of ribosomal translation. Readthrough is a constitutive process by which an amino acid, carried by a tRNA, is incorporated into the nascent polypeptide chain at the PTC instead of premature translation termination, enabling the expression of full-length proteins. Readthrough agents for nonsense mutations are small molecules that bind to the decoding center of the ribosome stimulating this PTC readthrough and facilitating near cognate aminoacyl-tRNA incorporation [13] (Table 1). Aminoglycosides are ribosome-binding antibiotics, and they were the first to be studied in cystic fibrosis for their nonsense readthrough properties [14]. In short clinical studies, none of the tested aminoglycosides restored enough functional CFTR protein to allow a prolonged clinical benefit [15,16]. Moreover, their strong oto- and nephrotoxicity prevent their long-term use in patients.

Ataluren is a non-aminoglycoside small molecule with readthrough properties in vitro that has failed to show in vivo efficacy in phase 3 trials in cystic fibrosis [17]. More recently, ELX-02, a small eukaryotic ribosomal selective glycoside, derived from the initial glycoside core, has shown promise as a PTC readthrough therapeutic and is currently investigated in phase 2 trials in patients with cystic fibrosis caused by the *G542X* mutant [18]. 

However, the efficacy of such therapeutics is still limited for several reasons [19,20]. First, the response to readthrough compounds depends on the PTC identity (UGA > UAG >> UAA) and its surrounding nucleotide sequence (a cytosine at the + 4 nucleotide is known to increase the response). Second, NMD efficiency fluctuates, which results in variable levels of intracytoplasmic transcripts. NMD may vary according to the position of the PTC in the mRNA, as mutations in the last exon or those located less than 50 nucleotides from the 3′ exon–exon junction are not subjected to NMD. Moreover, some mutations within *CFTR* may be less sensitive to NMD than others. Different strategies are studied to inhibit NMD and enhance mRNA substrates for readthrough agents. Some approaches showed a significant rescue of CFTR protein in vitro [21]. However, NMD plays important roles in cellular physiology, and whether NMD inhibition is safe and well-tolerated in patients remains to be shown. Finally, and most importantly, the nature of the amino-acid inserted by readthrough is variable and influences the resulting activity of the recoded channel, which may eventually be increased by CFTR modulators [22,23]. For example, for the C terminal *W1282X* mutation, the inhibition of NMD led to an increased abundance of the shorter transcript, which retains a very modest residual activity that can be enhanced by CFTR modulators [24]. For PTCs located in the middle of CFTR, such as *G542X*, drug-induced readthrough appears to be the main strategy, as truncated proteins are non-functional [20]. However, the encoding of a near-cognate amino acid may generate a missense mutation at the PTC, which may have deleterious effects on protein folding, trafficking, and function. This questions whether readthrough strategy should also favor amino acid incorporation that preserves channel function, as already reported by Pranke et al. [22]. 

## 4. RNA-Based Therapies

**Figure 1:**
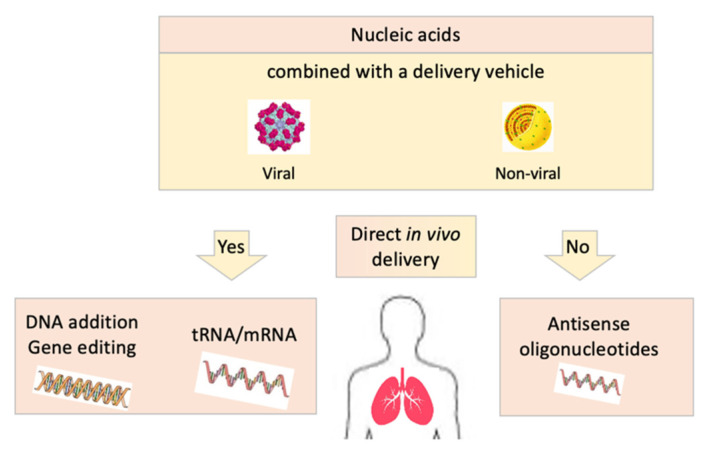
Different strategies of direct in vivo delivery of nucleic acid-based therapies in cystic fibrosis: modified nucleic acids combined or not to a delivery vehicle are administered in vivo in patients.

There are several types of RNA, of which three are under investigation for their putative therapeutic use in cystic fibrosis: transfer RNA (tRNA), messenger RNA (mRNA) and small antisense RNA molecules called antisense oligonucleotides (Table 1 and Figure 1). Depending on the type of RNA used, distinct subsets of patients with cystic fibrosis may benefit from it. 

It is estimated that restoring 5% of wild-type CFTR mRNA in the cytosol is enough to protect from severe respiratory disease, the threshold to limit evolution later in life being probably comprised between 10 and 30% [50]. This should be the benchmark for such strategies. 

Transfer RNAs couple with mRNA and ferry the amino acids composing the proteins. Anticodon-engineered suppressor tRNAs (ACE tRNAs) may be beneficial for patients with cystic fibrosis bearing nonsense mutations. They are designed to carry a nonsense-suppressing anticodon to address PTCs and to introduce the correct amino acid in the elongating peptide [25]. They are recognized by the endogenous translation cellular machinery, including the aminoacyl-tRNA synthetase that charges the ACE-tRNA with their cognate amino acid and the eukaryotic elongation factor 1α (eEF-1α), which delivers the charged tRNA to the ribosome. This approach has been successful in vitro at promoting the readthrough of stop codons in cystic fibrosis airway epithelial cells [26], and it has the advantage of incorporating the correct amino acid, leading to a normal functional protein. Genome-wide transcriptome ribosome profiling of cells expressing ACE-tRNA at levels which repair PTC indicate that there are limited interactions with translation termination codons [26]. However, studies are needed to investigate potential interaction with the cellular translation machinery. Moreover, suppressor tRNAs are macromolecules that are not readily taken up by cells, and they require effective delivery technologies. As delivery is one hurdle common to many nucleic acid-based therapies, it will be discussed later in this review. 

Messenger RNA is transcribed from DNA in the nucleus, and it is then translated by ribosomes in the endoplasmic reticulum into a protein. The delivery of CFTR-encoding mRNA into target cells, or mRNA addition, would theoretically lead to the translation of mRNA and eventually, the synthesis of a normal CFTR protein [27]. This therapy would be mutation-agnostic and benefit all patients with cystic fibrosis. Exogenous nucleic acids need to be modified to reduce immunogenicity and increase stability. But manufacturing and modifying RNA is easier than DNA [28]. Moreover, in contrast to DNA-based therapy, the difficult step of crossing the nuclear membrane is not necessary [29]. RNA addition is also expected to offer high levels of protein production but, as the half-life of mRNA is only a few hours, repeated delivery will be required. A delivery vehicle is needed for mRNAs and because of expected repeated administrations, a non-viral vector will probably be the best option [30]. The delivery of CFTR mRNA in vitro has shown increased CFTR expression and rescue of chloride transport in bronchial epithelial cells [31], and preclinical research is very active in this field. This was potentiated by the CRISPR nuclease strategy (see below). The CRISPR approach is not agnostic to genotype, since each CRISPR drug would need to be targeted to the mutation site. Interim results from an ongoing phase 1/2 placebo-controlled trial in cystic fibrosis showed that nebulisation into the lungs of patients with cystic fibrosis once a week over 5 weeks of CFTR mRNA packaged into delivery vehicles based on lipids was safe and well tolerated. However, so far, no increase in the respiratory function was observed (https://investors.translate.bio/news-releases/news-release-details/translate-bio-announces-results-second-interim-data-analysis accessed on 15 September 2021). The effect duration of this mRNA strategy needs investigation. In theory, the CFTR protein is only produced for a short period of time, meaning that frequent administration would be needed. This may lead to toxicities from high doses of the vehicle carrying the CFTR mRNA. On the other hand, the CFTR gene is transcribed at low levels, and the mature protein product can be stable for extended periods of time, with a half-life of >15 h after reaching the plasma membrane [51]. 

In addition to the above RNA approaches, another option is to develop small antisense RNA-like molecules or antisense oligonucleotides (ASOs). They are synthetic oligonucleotides chemically modified to bind to target RNA for direct RNA restoration or the correction of *CFTR* splicing mutations. One advantage of this technology is that, as ASOs are small molecules, no delivery vehicle is needed for their administration. Eluforsen is a 33-oligonucleotide that was designed to hybridise to CFTR mRNA at the *p.Phe508del* encoding site and to restore CFTR function. The *p.Phe508del* mutation is one of the most common *CFTR* mutations. It consists of a deletion of three nucleotides, leading to the loss of phenylalanine at position 508 (*p.Phe508del*) in the protein. Eluforsen was shown to improve CFTR function in cell and animal models of *p.Phe508del*-CFTR-mediated cystic fibrosis [32]. Intranasal administration of eluforsen in patients homozygous for the *p.Phe508del* mutation in a 4-week open-label trial showed improvements in CFTR function in nasal epithelium [33]. A subsequent phase 1 trial showed that the pulmonary nebulisation of eluforsen over 4 weeks was safe and well-tolerated, but no change in respiratory function was observed [34], and no further studies are planned to evaluate eluforsen in cystic fibrosis. Another therapeutic use of ASOs in cystic fibrosis could be for mutations involving aberrant exon splicing. RNA splicing is the process by which introns are removed from precursor mRNA. Splicing mutations disrupt intronic or exonic splicing motives and lead to aberrant mRNA and non-functional protein by creating or abolishing canonical splice sites, commonly leading to skipping over the exon [52]. There is another group of mutations altering regulatory splicing motives throughout the gene, leading to variable levels of both aberrantly and correctly spliced transcripts from these mutated alleles. This group includes the splicing mutation *3849* + *10Kb C* > *T*, which is associated with reduced amount of normal CFTR. A correlation was found between the amount of correctly spliced CFTR transcripts and lung function, which highlights the potential of splicing modulation as a therapeutic approach [53]. This strategy proved efficient in primary respiratory cells carrying the *3849* + *10Kb C* > *T* variant [35]. ASOs were shown to modulate splicing in cells with various *CFTR* splicing mutations and to improve CFTR activity in bronchial epithelial cells [36,37]. They act by inhibiting or activating specific splicing events by a steric blockade of the recognition of specific splicing elements, and thus, they prevent the recruitment of effectors to these sites. No evaluation in a clinical trial of ASOs for *CFTR* splicing mutations has been undertaken so far. Nevertheless and importantly, ASO-based drugs modulating splicing are already approved for spinal muscular atrophy and Duchenne muscular dystrophy and provide remarkable improvements. This highlights the potential of such therapies in the cystic fibrosis field.

## 5. DNA-Based Therapies: Gene Therapy and Gene Editing 

Gene therapy consists of delivery of *CFTR* cDNA with regulatory components into cells resulting in normal CFTR protein synthesis alongside the constitutive abnormal CFTR protein [33]. To be successful, gene therapy needs to use an effective delivery technology or vector that allows the *CFTR* cDNA to reach and enter airway epithelial cells, and then be transcribed and translated to express the normal CFTR protein (Table 1 and Figure 1). A clear advantage of gene therapy is that it is not mutation-specific: one type of treatment would benefit all patients. Soon after the *CFTR* gene was identified in 1989, major gene therapy research programmes were conducted with many different approaches involving viral and non-viral vectors. Multiple clinical trials were run. Some level of CFTR correction has been shown in vitro in airway epithelial cells and rectal organoids, and in vivo in patients with a good safety profile. But, three decades later, gene therapy has yet to be a valid therapeutic approach for patients with cystic fibrosis. The delivery of normal *CFTR* cDNA into airway cells in humans has proven more challenging than originally anticipated. Several hurdles have been identified, such as finding the appropriate plasmid DNA model, overcoming natural barriers like airway mucus, targeting and entering highly differentiated airway epithelial cells with a low dividing rate, and limiting immune responses. As with nucleic acid-based therapies, one of the main issues is identifying the most effective vector that can combine the *CFTR* cDNA and improve its delivery to target cells [38,39]. 

Gene editing exploits cellular DNA repair pathways (Table 1 and Figure 1). It repairs mutations in the *CFTR* gene and is mutation-specific. It is based on the delivery into target cells of both the correct version of the *CFTR* DNA sequence and a nuclease. The nuclease causes a break in the DNA near the mutation site, and this break triggers recombination and DNA reparation. Different nucleases can be used such as zinc finger nucleases (ZFNs), transcription activator-like effector nucleases (TALENs) and clustered regularly interspersed palindromic repeats (CRISPR)/CRISPR-associated nuclease 9 (Cas9). CRISPR/Cas9 targets a specific chromosomal site by guide RNAs. Its simple use, low cost and anticipated low risk of off-target breaks has made CRISPR/Cas9 the main approach of gene editing studies in cystic fibrosis [40,41]. The first proof-of-concept study was published in 2013 and showed repair of the *p.Phe508del* mutation by gene editing in intestinal organoids [42]. Many other in vitro studies have followed [43]. No clinical study in cystic fibrosis has been undertaken yet with this approach, which requires the use of effective vectors. This new approach is blooming, and clinical trials are underway to evaluate gene editing in the treatment of diseases like cancer and sickle cell disease [44]. 

## 6. Cell-Based Therapies

**Figure 2:**
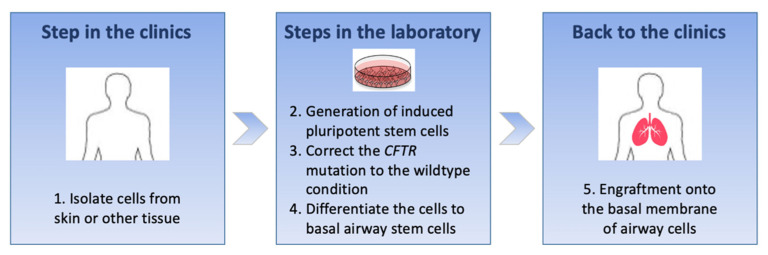
Main steps for cell-based therapy in cystic fibrosis: it is an ex vivo approach wherein patient’s cells are collected and modified in vitro to obtain corrected basal airway stem cells and then transferred back into the patient.

The ultimate goal for DNA-based therapies in cystic fibrosis is to target and correct enough stem cells with a single administration to populate the airways and restore CFTR function throughout the patient’s lifespan. To achieve this, the gene editing of airway stem cells would be the aim. But airway stem cells are deeply hidden near the basement membrane and are not easy to target in vivo [38]. The recent description of induced pluripotent stem cells (iPSCs) paved the way for a new cell-based therapy approach using ex vivo gene editing. iPSCs are cells coming from fully differentiated cells, such as fibroblasts or cutaneous cells, that are reprogrammed to resemble the least differentiated embryonic stem cells. They are then reprogrammed again to differentiate into specific lineages such as airway epithelial cells. iSPCs can proliferate rapidly and indefinitely [54]. iPSC-based ex vivo gene editing may be a future approach to treat cystic fibrosis [55]. Genetic correction with TALENs and the CRISPR/Cas system of fibroblast-derived iPSCs bearing the *p.Phe508del* mutation and differentiated to form airway epithelial cells was reported [45] (Table 1 and Figure 2). 

It will be a long road before this cell-based therapy approach can become a reality, and many different steps need to be considered. First, cells from a patient with cystic fibrosis are isolated and manipulated in the laboratory to reprogramme them into iPSCs; then, the *CFTR* mutation is converted to the wildtype status, and finally, the corrected iPSCs are turned into basal airway stem cells which have the capacity to differentiate into all cell types of pseudostratified airway epithelium [46]. The last step is to engraft corrected basal cells onto the patient’s basement membrane of the airway epithelium to achieve an autologous graft and replenish the airways with a fully CFTR-corrected airway epithelium [47]. Major challenges are the need to derive a pure population of airway epithelial cells from iPSCs in sufficient numbers to engraft in human airways, engrafting these cells in airways in a safe and effective manner and making sure that the corrected cells are free of integrations and somatic mutations [48]. It is estimated that as many as 60 million regenerative cells may be required to treat a cystic fibrosis patient with cell therapy [49]. Moreover, the engraftment of cells adjacent to the basement membrane would require disruption of the epithelial cell layer by transient injury, which may be deleterious. Last but not least, the safety of this strategy remains the key question, as reprogramming, expanding and editing cells increase the probability of tumorigenicity [48]. 

## 7. Delivery Vectors

Most RNA- and DNA-based therapies require the use of a delivery vehicle or a vector because those molecules have high negative charges, which make them unable to cross the cell and the nuclear membrane. Delivery vehicles for gene therapy fall broadly into two categories: viral and non-viral vectors. 

Viral vectors are usually more efficient, and adenoviruses or adeno-associated viruses have a natural tropism for airway cells and can cross the mucus barrier. Several clinical trials in cystic fibrosis using these viral vectors failed to show a sufficient level of *CFTR* transgene expression. Pre-existing and induced immune responses to the viral vector limited its efficacy. New modified viral vectors are being studied [39]. Current strategies include the use of a simian-based lentiviral vector, pseudotyped with Sendai virus fusion protein and hemagglutinin/neuraminidase envelope proteins that exhibit an efficient transduction of human airway cells in vitro and in vivo in murine lung epithelium with a 2 year-long expression [56].

Non-viral formulations are simple chemical structures that are less likely to induce immune responses. Liposomes are artificially created vesicles with a lipid bilayer membrane that can encapsulate and deliver nucleic acid to cells. They have been shown to be well-tolerated in several clinical trials in cystic fibrosis, but their efficacy was limited. Other nonviral vectors such as lipid nanoparticles or exosomes are being worked on [47]. Exosomes are extracellular vesicles secreted by cells and carrying proteins, lipids or the mRNA of neighbouring or distant cells [57]. They have been demonstrated to deliver CFTR protein and mRNA and to correct channel function [58].

## 8. Conclusions

Proteic therapy for cystic fibrosis will mean a completely different disease and life perspective for many patients. But for those who bear *CFTR* mutations not eligible for CFTR protein therapy, new approaches need to be pursued to propose a disease-modifying treatment for all patients. For those who bear rare mutations that might be responsive to current CFTR modulators, new ways of evaluating drugs in very scarce population are worked on. For those who bear mutations that do not produce any CFTR protein, mutation-specific or mutation-agnostic therapies are being developed. Most of these therapies are still in a preclinical research state, and before they can be safely translated to patients, many challenges will have to be solved. 

## Figures and Tables

**Table 1:** Summary of the different therapeutic approaches to treat patients with cystic fibrosis bearing mutations non-responsive to current CFTR modulators (see text for details).

Therapeutic Approaches	Mechanism of Action	Mutation Specificity	Issues to Overcome	Examples of References
**Readthrough** **agents**	Incorporation of an amino acid instead of a H_2_O molecule, leading to premature translation termination	Nonsense mutations	Nonsense-mediated decay reduces mRNA to act upon; amino acids altering the neoformed protein function can be incorporated; potential theoretical effect on the terminal stop codon; repeat administration needed	[13,14,15,16,17,18,19,20,21,22,23,24]
**Engineered** **transfer RNA**	Carries a nonsense suppressing anticodon to address the premature translation termination codon	Nonsense mutations	Need for an effective delivery vehicle to deliver to target cells and overcome natural barriers; repeat administration needed	[25,26]
**mRNA addition**	Addition of the correct CFTR-mRNA and synthesis of the CFTR protein	Mutation-agnostic	Need for an effective delivery vehicle to deliver to target cells and to overcome natural barriers; repeat administration needed	[27,28,29,30,31]
**Antisense oligonucleotides**	Oligonucleotides chemically modified to bind and restore target RNA	Mutation-specific	Need to deliver to target cells and to overcome natural barriers; repeat administration needed	[32,33,34,35,36,37]
**DNA addition**	Addition of the correct *CFTR*-encoding cDNA and synthesis of the CFTR protein	Mutation-agnostic	Need for an effective delivery vehicle to deliver to target cells and to overcome natural barriers, including the nuclear membrane	[38,39,40]
**Gene editing**	Repair of the mutation by the delivery of a nuclease and the correct *CFTR* cDNA sequence guide	Mutation-specific	Need for an effective delivery vehicle to deliver to target cells and to overcome natural barriers, including the nuclear membrane	[41,42,43,44]
**Cell-based** **therapies**	Gene editing of airway epithelial stem cells for later engraftment onto the airway basal membrane	Mutation-specific	Need to control the phenotype of corrected cells: type of cells, free of new mutation; safe and effective manner to engraft corrected cells into airways	[45,46,47,48,49]

## Data Availability

Not applicable.
